# Comparative Assessments of Seismic Drift Demands and Dispersions in RC Piloti Buildings with Eccentric Cores Using CSM–MCS and IDA

**DOI:** 10.3390/ma19214723

**Published:** 2026-11-06

**Authors:** Chang-Geun Cho, Hyeon-Jin Lim, Hyoung-Hun Yeom, Hyung-Joo Moon

**Affiliations:** 1 Department of Architectural Engineering, Chosun University, Gwangju 61452, Republic of Korea; chocg@chosun.ac.kr; 2 Department of Construction Convergence, Mokpo Science University, Mokpo 58644, Republic of Korea; 3 Department of Structural Design, I’st Structure Solution, Seoul 05836, Republic of Korea; sunsea088@naver.com; 4 Department of Architecture, Dongshin University, Naju 58245, Republic of Korea; jjangmoonhj@naver.com

**Keywords:** reinforced concrete, piloti building, core eccentricity, capacity spectrum, incremental dynamic analysis, empirical distribution, response dispersion, record sensitivity

## Abstract

**Highlights:**

- Mean EC2-to-CC1 drift ratios are 6.01–6.45 for CSM–MCS and 1.39–1.55 for IDA.
- Configuration rankings vary with the statistic and listed motion groups.
- Minimum common-scale mismatch factors are 2.93 for means and 3.95 for medians.
- Absolute demand and relative dispersion should be interpreted jointly.

**Abstract:**

Seismic drift comparisons in reinforced-concrete (RC) piloti buildings depend on core configuration, response statistic and ground-motion composition. This study compares the capacity spectrum method coupled with Monte Carlo simulation (CSM–MCS) and incremental dynamic analysis (IDA) for two nominal central-core (CC1, CC2) and two eccentric-core (EC1, EC2) configurations at intensity coordinates of 0.598 and 0.702 g. The static branch varies material strengths under fixed 5% demand damping; the dynamic branch varies motions at nominal strengths. Mean EC2-to-CC1 ratios are 6.01–6.45 for CSM–MCS and 1.39–1.55 for IDA. At 0.702 g, the IDA 95th percentile is 0.745% for CC1 and 0.630% for CC2, reversing their mean ordering. Common-record comparisons retain the baseline mean ordering, while omitting the listed Loma Prieta group reverses the two intermediate configurations. A common proportional scale leaves minimum worst-case mismatch factors of 2.93 for means and 3.95 for medians. The integrated comparison shows how response scale, distributional shape and record composition affect assessment judgments within the two conditional analysis populations.

## 1. Introduction

Reinforced-concrete (RC) piloti buildings provide an open ground story for parking and access beneath comparatively stiff upper stories. The resulting discontinuity in lateral resistance can concentrate deformation in the ground-story columns. Observations after the 2017 Pohang earthquake and numerical studies of piloti-column retrofit establish the relevance of this demand concentration [1,2]. European work on soft-story RC retrofit provides related evidence on the interaction between upper-story infills and the open ground-story frame [3]. An eccentric core introduces a further configuration variable into the assessment of these systems.

Core eccentricity couples translation and torsion, while inelastic stiffness redistribution can alter that coupling. Studies of nonlinear torsional amplification and pushover assessment show that initial eccentricity alone does not characterize the nonlinear response of asymmetric buildings [4,5]. Directionally normalized eccentricities can also follow different orders across configurations. Both directional descriptors are therefore needed when interpreting the response of discrete core arrangements.

Material properties, nonlinear idealization and ground-motion characteristics introduce different forms of variability. Recent RC fragility studies consider multiple uncertainty sources, material-property effects and ground-motion characteristics [6,7,8], while prediction approaches require a clearly defined response target and a consistent numerical population [9]. Dolšek combined structural-model and record variability in extended incremental dynamic analysis (IDA) of an RC frame [10]. North American research has used nonlinear dynamic assessment to study collapse of ductile RC moment frames [11]. These studies establish the importance of relating response statistics to the structural system, input population and performance objective being assessed.

The capacity spectrum method (CSM) relates nonlinear static capacity to demand in equivalent single-degree-of-freedom (SDOF) coordinates [12,13]. IDA traces response under scaled ground motions [14]. The represented variability depends on implementation. FRACAS (FRAgility through Capacity spectrum ASsessment), for example, includes record variability in a capacity-spectrum framework [15]. The CSM coupled with Monte Carlo simulation (CSM–MCS) considered here varies material strengths under prescribed spectra, whereas IDA varies ground motions at nominal strengths. Their dispersions describe different conditional populations.

A comparison based only on mean drift and standard deviation (SD) can overlook distributional differences. The coefficient of variation (COV) normalizes spread by the mean, so a reduction in COV need not indicate smaller absolute demand. A mean-based configuration ranking need not persist for the median or an upper percentile. Motion-suite composition can also influence closely spaced configuration means. These issues motivate an assessment that retains both the distribution and the identity of the response entries.

This article examines three questions. First, how do central statistics, absolute and relative dispersion, and empirical percentiles characterize the four configuration datasets? Second, how do the contrasts between implementations vary with intensity, and can one proportional scale represent them? Third, how sensitive are the IDA comparisons to listed record groups and common-record selection? The contribution is an integrated evaluation of statistical ordering, distributional change and record influence, supported by a common-scale mismatch bound. Table 1 distinguishes the adopted analysis basis from the additional comparisons developed here.

Section 2 defines the analysis design, archetype and statistical calculations. Section 3 presents demand, distributional and record-sensitivity results. Section 4 develops their interpretation and assessment implications, and Section 5 states the conclusions. Appendix A identifies the listed motion suite, Appendix B provides the reported response diagrams, and Appendix C documents the correspondence and eligibility conditions for the numerical comparisons.

## 2. Materials and Methods

### 2.1. Analysis Design and Numerical Basis

The four nominal building configurations and the underlying CSM–MCS and IDA analyses are described in [16]. They are designated central-core configurations 1 and 2 (CC1, CC2) and eccentric-core configurations 1 and 2 (EC1, EC2). This article develops an entry-level comparison of 16 configuration–procedure–intensity cases at 0.598 and 0.702 g, with additional numerical results across seven CSM–MCS and eight IDA coordinates. The additional analyses integrate empirical distributions, record composition, influence and proportional consistency within these structural-response ensembles.

The comparison target is the documented response ensemble for each conditional implementation. Statistical matching assigns the center-2/Cp and eccentric-1/E1 response files to EC1 and CC2, respectively, while their physical geometry-to-response connection remains unverified. Differences between some summary values and direct exports, and exceptions in input-preparation sheets, are documented in Appendix C. Accordingly, the distributional and influence findings are conditional descriptive results; they do not establish a verified configuration-specific torsional contribution or structural reliability. Supplementary File S1 provides the corresponding statistical summaries.

Statistics are calculated from individual numerical entries before rounding. CSM–MCS uses the 50 indexed sample columns, excluding the separate nominal-reference column. This corrects the earlier low-intensity CC2 aggregation that included that reference entry. IDA uses nonmissing values in each case, giving *n* = 21 or 22 at the two principal coordinates. Missing or omitted values are not replaced with zero, and formula-linked values are identified in Appendix C.

### 2.2. Building Geometry and Core Layouts

**Figure 1:**
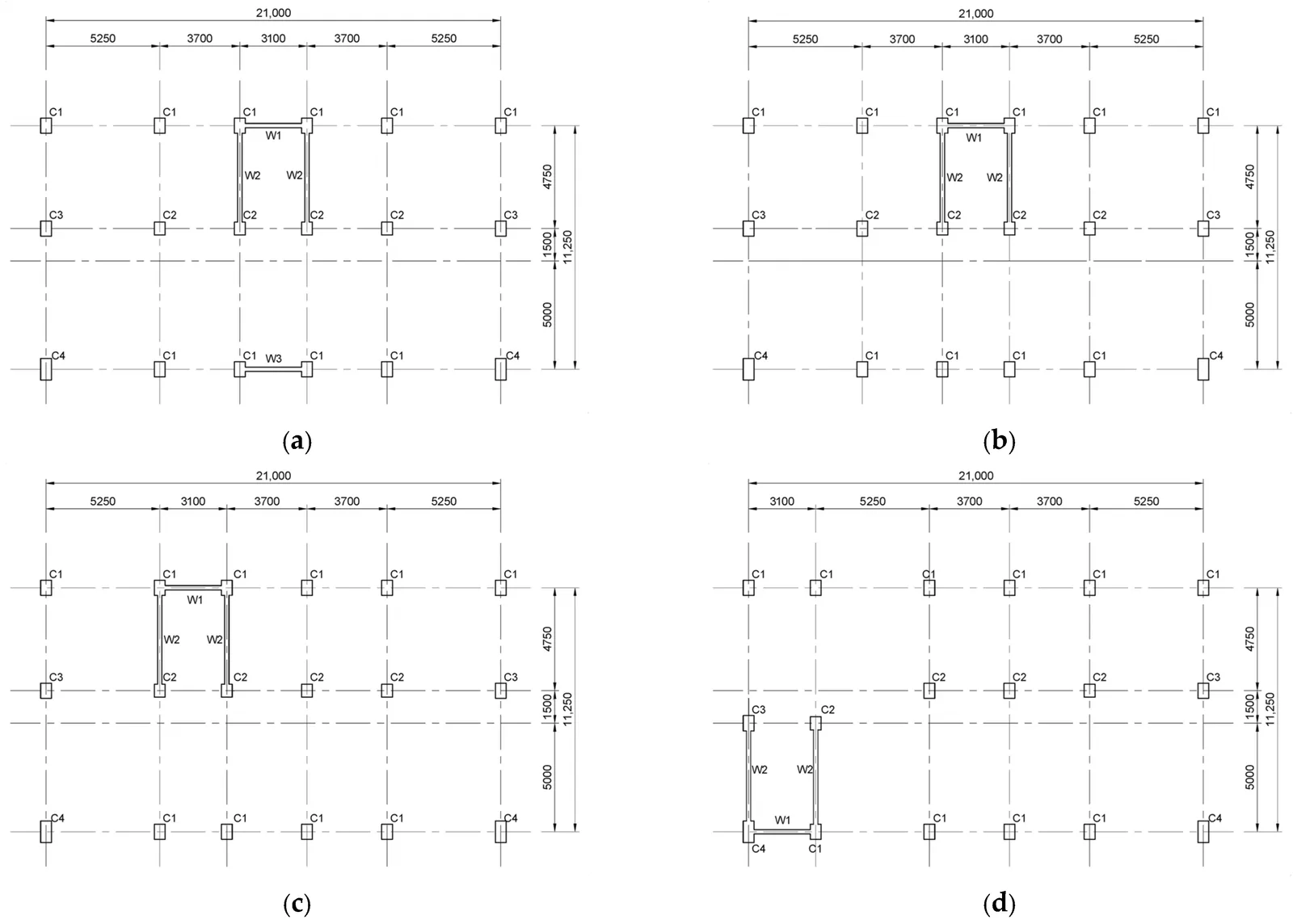
Structural plans and core locations for (**a**) CC1, (**b**) CC2, (**c**) EC1 and (**d**) EC2. Overall dimensions are 21.00 m × 11.25 m. Adapted from [16]. C1–C4 and W1–W3 identify column and wall member types, respectively; plan dimensions are in millimeters.

**Figure 2:**
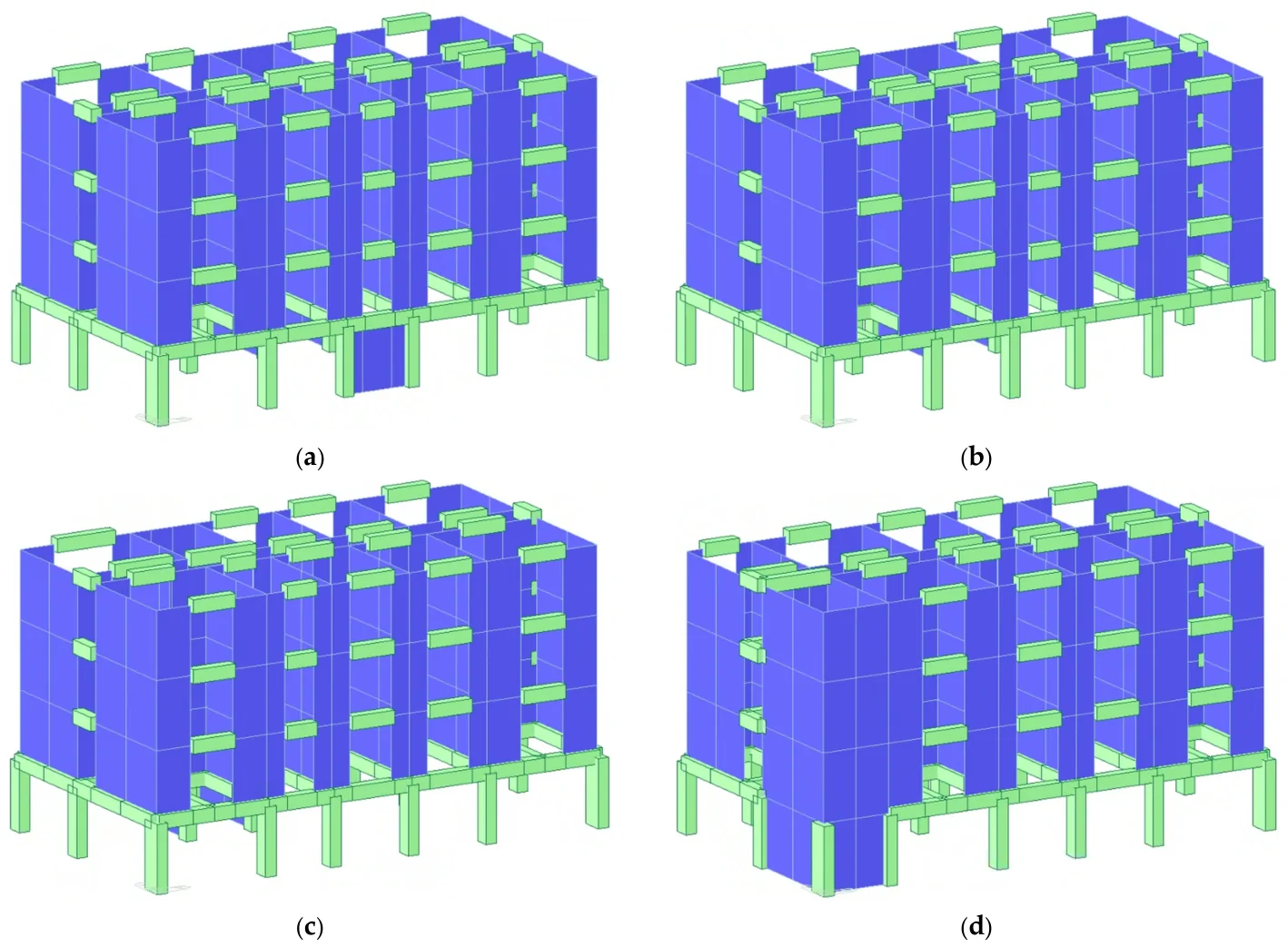
Numerical models of the four-story piloti archetype for (**a**) CC1, (**b**) CC2, (**c**) EC1 and (**d**) EC2. Story height is 3.0 m. Adapted from [16]. Green denotes frame members (beams and columns), and blue denotes wall elements.

The archetype represents a surveyed four-story RC piloti building in Pohang, Republic of Korea. Its plan measures 21.00 m × 11.25 m, with 3.0 m story heights and a total modeled height of 12.0 m. The ground-story core arrangement varies, while principal geometry, wall quantity, member sizes, mass and story heights are reported as common modeling controls [16]. Figure 1 and Figure 2 reproduce the nominal plans and three-dimensional models. Table 2 follows the geometric labels; the response-label convention in Section 2.1 remains applicable.

Let *e*_X_ and *e*_Y_ denote absolute offsets between the centers of mass and rigidity, *K*_X_ and *K*_Y_ the translational stiffnesses in the X and Y directions, and *K*_θ_ the torsional stiffness. The stiffness-based torsional radii and normalized offsets are defined by

(1) $$r_{\mathrm{eX}}=\sqrt{\frac{K_{\theta }}{K_{X}}},\;r_{\mathrm{eY}}=\sqrt{\frac{K_{\theta }}{K_{Y}}}$$

(2) $$R_{\mathrm{eX}}=\frac{e_{Y}}{r_{\mathrm{eX}}},\;R_{\mathrm{eY}}=\frac{e_{X}}{r_{\mathrm{eY}}}$$

The perpendicular offset enters each ratio because a translational action produces a torsional moment through that offset. These ratios differ from eccentricity normalized by plan dimensions and from drift-based code irregularity indices. The nominal EC2 geometry has the largest *R*_eY_, whereas its *R*_eX_ is smaller than those of CC2 and EC1. The four cases are discrete configurations rather than successive values of one scalar eccentricity.

### 2.3. Structural Idealizations and Numerical Procedures

The three-dimensional models follow the Korean seismic design standard for buildings (KDS 41 17 00:2022), with survey-informed member dimensions and dead loads and code-based live loads [16,17]. CSM–MCS represents beams, columns and walls by concentrated plastic hinges with Federal Emergency Management Agency (FEMA) 273-type backbones [18]. IDA uses beam hinges, column and wall fiber elements, a modified Kent–Park concrete representation [19], and an energy-based cyclic model with a FEMA 356-type envelope [16,20]. Ibarra and coauthors provide the broader context for strength and stiffness deterioration modeling [21]. These idealizations are retained as distinct conditions of the two procedures. Table 3 summarizes the analysis conditions for both implementations.

The nonlinear time-history analyses were performed using MIDAS Gen (version 9.5.0; MIDAS IT Co., Ltd., Seongnam, Republic of Korea). The IDA fiber sections described in [16] integrate uniaxial concrete and steel responses under a plane-section assumption, allowing axial force and bending to interact. The modified Kent–Park representation distinguishes confined and unconfined concrete [16]. Nominal concrete and steel strengths are 21 and 400 MPa. The described dynamic procedure uses 5% Rayleigh damping, Newmark average-acceleration integration with *β*_N_ = 1/4 and *γ*_N_ = 1/2 [22], and modified Newton–Raphson iteration.

For CSM–MCS, pushover curves are converted to acceleration–displacement response-spectrum (ADRS) coordinates and idealized by an energy-equivalent bilinear curve [16]. The initial line passes through the point at 60% of the effective yield force; the terminal point is the earlier of the selected target-displacement and maximum-base-shear points. The yield intersection is adjusted to approximately balance the areas between the capacity and idealized curves. The probabilistic branch intersects these capacities with prescribed 5%-damped demand spectra. This fixed-damping implementation differs from iterative effective-damping CSM procedures [12,13]. The capacity spectra and bilinear idealizations are shown in Appendix B.

### 2.4. Intensity Measure and Spectrum Convention

The nominal intensity measure (IM) is *S*_a_(*T*_1_, 5%), the 5%-damped elastic spectral acceleration at a fundamental-period reference *T*_1_ [16]. The reported spectra assume seismic zone I and site class S4. Table 4 gives the seven static coordinates and their return-period labels. The two principal comparison coordinates are numerical IM labels; this study does not introduce a site-specific hazard calibration.

**Figure A1:**
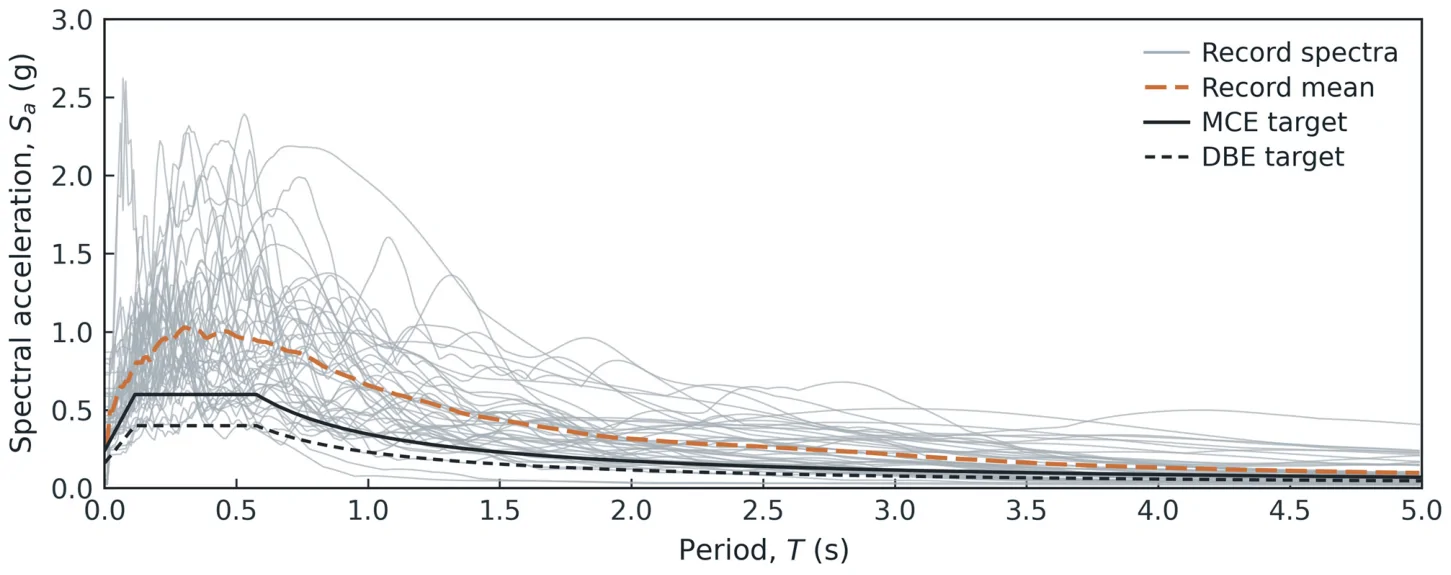
Record-component spectra and their stored mean with maximum considered earthquake (MCE) and design-basis earthquake (DBE) target curves, redrawn from the numerical spectrum coordinates associated with Figure 5.11 in [16]. DBE is two-thirds of the prescribed MCE target. Applied-input correspondence is specified in Appendix C.

The reported IDA procedure uses amplitude scaling and a suite-spectrum comparison with the maximum considered earthquake (MCE) target over 0.2*T*_1_–1.5*T*_1_ [16,23]. Amplitude scaling changes acceleration amplitudes without matching the waveform spectrum point by point. Figure A1 reproduces the spectrum comparison for context.

Two input-preparation sheets record *T*_1_ values of 0.2065 and 0.2067 s and separate target-*S*_a_ and scale-factor cells. Their acceleration-function columns are identical while target settings differ. These settings are distinguished from verified period assignments and applied scaling for the final configurations. The IM-folder and motion-identity conditions are specified in Appendix C.

### 2.5. Material Sampling

The reported material model assigns normal concrete compressive strength and lognormal steel yield strength, with arithmetic parameters in Table 5 [16]. These COVs are modeling assumptions. Material and motion variability describe different uncertainty populations [24]. The reported design uses Latin hypercube sampling (LHS) [25], with 50 indexed entries per configuration.

Postprocessing uses the 50 indexed response columns. Smaller-*n* files do not establish independently traceable simulation samples and are excluded from convergence evidence. Formula-linked cells are identified in Appendix C. The analyses below assess distributions and influence without assigning a replicated-LHS or independent-draw Monte Carlo confidence interpretation.

### 2.6. Listed Motion Suite and Statistical Units

The selection list contains 11 pairs from the FEMA P695 near-field pulse subset and 11 pairs from its far-field set, giving 22 listed pairs and 44 component functions [16,26]. Appendix A supplies the intended event–station metadata, Pacific Earthquake Engineering Research Center (PEER) Next-Generation Attenuation (NGA) record sequence numbers (RSNs), magnitudes, average shear-wave velocity in the upper 30 m (*V*_s30_), and closest-rupture distances *R*_rup_. Magnitudes are 6.5–7.6. This mixed benchmark list is not a hazard-consistent representation of the magnitude-5.4 Pohang event.

The response tables contain one value per numbered record case, with 21 or 22 nonmissing entries at the principal coordinates. The 44 component functions are not treated as 44 independent drift observations. Listed pairs 1–11 and 12–22 define the near and far groups. Event-omission calculations use the event names in the selection list, including repeated stations from one earthquake; this grouping is not an estimate of the number of independent earthquake observations. Input identity is qualified by Appendix C.

### 2.7. Response Definition and Comparison Indicators

The engineering demand parameter (EDP) is the peak interstory drift ratio, expressed as a percentage. Its general dynamic definition is

(3) $$r_{\mathrm{dyn}}\;(\%)=100\;\underset{i,p,d,t}{\max}\;\frac{\left|u_{i,p,d}(t)-u_{i-1,p,d}(t)\right|}{h_{i}}$$

Here, *i* denotes a story, *p* a monitored plan position, *d* a horizontal direction and *h_i_* the story height. The adjacent-floor displacement difference is formed at each time before maximization. The static counterpart is evaluated at the reported performance point after equivalent-SDOF displacement is mapped to the building. Direct exports contain four story labels and separate AbsMax-X and AbsMax-Y values. The present calculations use the case entries under the correspondence conditions in Appendix C; the general definition does not establish a verified uniform monitoring-location or direction-combination rule for every case. Residual drift and column rotation are separate quantities.

Mean *μ*, median *m*, standard deviation *σ* and observed extrema are calculated on the percentage drift scale. The sample standard deviation uses divisor *n* − 1. A standard deviation of 0.189 denotes 0.189 percentage points. Relative dispersion and the mean–median contrast are

(4) $$\mathrm{COV}=\frac{\sigma }{\mu },\;Q=\frac{\mu }{m}$$

At the same configuration and numerical IM coordinate, the between-procedure ratios are

(5) $$R_{\mu }=\frac{\mu_{\mathrm{CSM}}}{\mu_{\mathrm{IDA}}},\;R_{\sigma }=\frac{\sigma_{\mathrm{CSM}}}{\sigma_{\mathrm{IDA}}}$$

The relative-dispersion ratio *R*_COV_ is the CSM–MCS COV divided by the IDA COV, equivalently *R_σ_*/*R_μ_*. Within each procedure, configuration amplification relative to CC1 is

(6) $$A_{\mu ,l}=\frac{\mu_{l}}{\mu_{\mathrm{CC}1}},\;A_{\sigma ,l}=\frac{\sigma_{l}}{\sigma_{\mathrm{CC}1}}$$

Finite amplification from the lower to the higher coordinate is

(7) $$G_{\mu }=\frac{\mu (0.702)}{\mu (0.598)},\;G_{\sigma }=\frac{\sigma (0.702)}{\sigma (0.598)}$$

COV amplification equals *G_σ_*/*G_μ_*. Ratios use full-precision statistics before rounding. These indicators characterize the complete conditional implementations and do not estimate isolated algorithm bias or separate material and motion variance fractions.

### 2.8. Empirical Distributions and Record Sensitivity

For *n* entries *d_r_* in one case, the empirical cumulative distribution is

(8) $$\hat{F}(x)=\frac{1}{n}\sum_{r=1}^{n}1(d_{r}\;\leq \;x)$$

The indicator equals one when *d_r_* does not exceed *x* and zero otherwise. *Q*_16_, *Q*_50_, *Q*_84_ and *Q*_95_ are sample percentiles obtained by linear interpolation. The zero-based position *t* = (*n* − 1)*p* defines the interpolation for percentile fraction *p*; neighboring one-based order positions are floor(*t*) + 1 and ceil(*t*) + 1. No parametric density fit is required.

Record-composition sensitivity is evaluated by omitting one numbered record or all records in one listed event. For a statistic *S*, relative change after event *e* is omitted is

(9) $$\Delta_{e}(S)=100\;[\;\frac{S_{-e}}{S}-1\;]$$

*S* is evaluated for the mean and COV; the same form is used for one-record omission. Original entries remain in the baseline ensembles. The largest record divided by the sum of case values gives its contribution to that sum. These are deterministic influence calculations, distinct from confidence intervals or deletion rules.

A separate comparison uses the intersection of nonmissing record IDs across the four IDA tables. The common sets contain 20 records at 0.598 g and 19 at 0.702 g. Baseline and event-omission comparisons use these same IDs, separating unequal counts from changes in the configuration ordering.

Finally, a common proportional relation is tested for the positive central estimates. Let *a_j_* and *b_j_* denote the same statistic from CSM–MCS and IDA in case *j*, and *r_j_* = *a_j_*/*b_j_*. For a positive scale *c*, *D*(*c*) is the largest multiplicative mismatch between *a_j_* and *c b_j_*. With *r*_min_ and *r*_max_ denoting the extreme case ratios,

(10) $$\begin{array}{c} D(c)=\max_{j}\;\{\;\max(\frac{r_{j}}{c},\;\frac{c}{r_{j}})\;\}\\ c^{*}=\sqrt{r_{\min}\;r_{\max}},\;D_{\min}=\min_{c>0}\;D(c)=\sqrt{\frac{r_{\max}}{r_{\min}}} \end{array}$$

The bound follows because *D*(*c*) is at least the larger of *r*_max_/*c* and *c*/*r*_min_. Equality gives c^*^; at that scale, all case ratios between the two extremes have a mismatch no larger than *D*_min_. The calculation is applied separately to means and medians and requires neither a distributional fit nor independent cases. It measures the consistency of a common proportional description; it is not a design correction or a physical prediction-error estimate.

**Figure 3:**
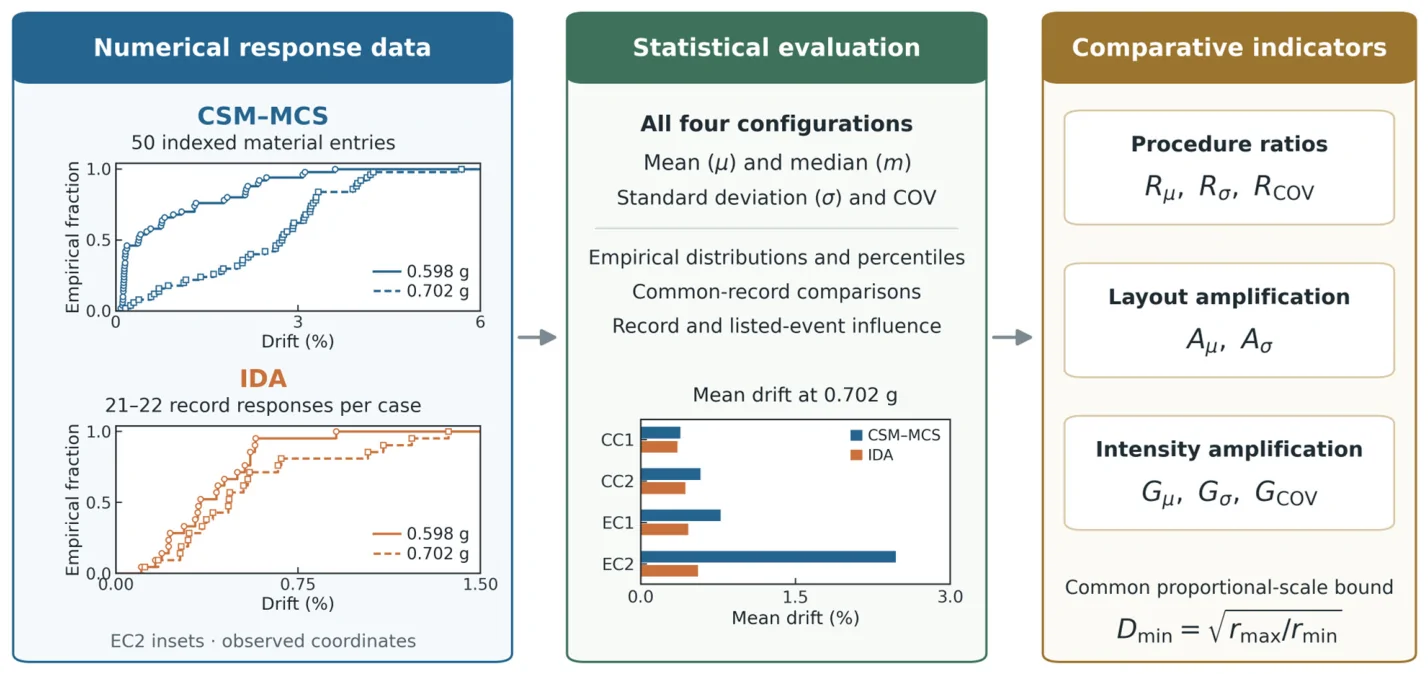
Three-stage comparative assessment framework: numerical response ensembles, statistical evaluation and comparative indicators. The EC2 insets show empirical cumulative distributions at 0.598 and 0.702 g using actual sample coordinates; the statistical calculations include all four configurations. The mean-drift inset uses full-precision values at 0.702 g. Analysis conditions are specified in Table 3.

Figure 3 summarizes the progression from the two conditional response ensembles to statistical evaluation and comparative indicators.

### 2.9. Use of Generative AI Tools

Generative artificial intelligence (AI) tools assisted with English-language editing, manuscript organization, graphical preparation, and development and computational checking of statistical postprocessing scripts. The original CSM–MCS and IDA building simulations were performed independently of these tools. The authors reviewed the AI-assisted text, equations, code and figures and checked the reported statistics against the numerical inputs. Tool information and the responsibility statement are provided in the Acknowledgments.

## 3. Results

### 3.1. Central Estimates and Observed Ranges

Table 6 and Table 7 summarize the individual-entry statistics of the two conditional response ensembles. For CSM–MCS, mean drift increases from 0.1458% for CC1 to 0.8765% for EC2 at the lower coordinate, and from 0.3831% to 2.4716% at the higher coordinate. EC2-to-CC1 mean amplification is 6.01 and 6.45, respectively. The corresponding IDA amplification is 1.39 and 1.55. The size of the configuration contrast therefore depends strongly on the conditional procedure.

**Figure 4:**
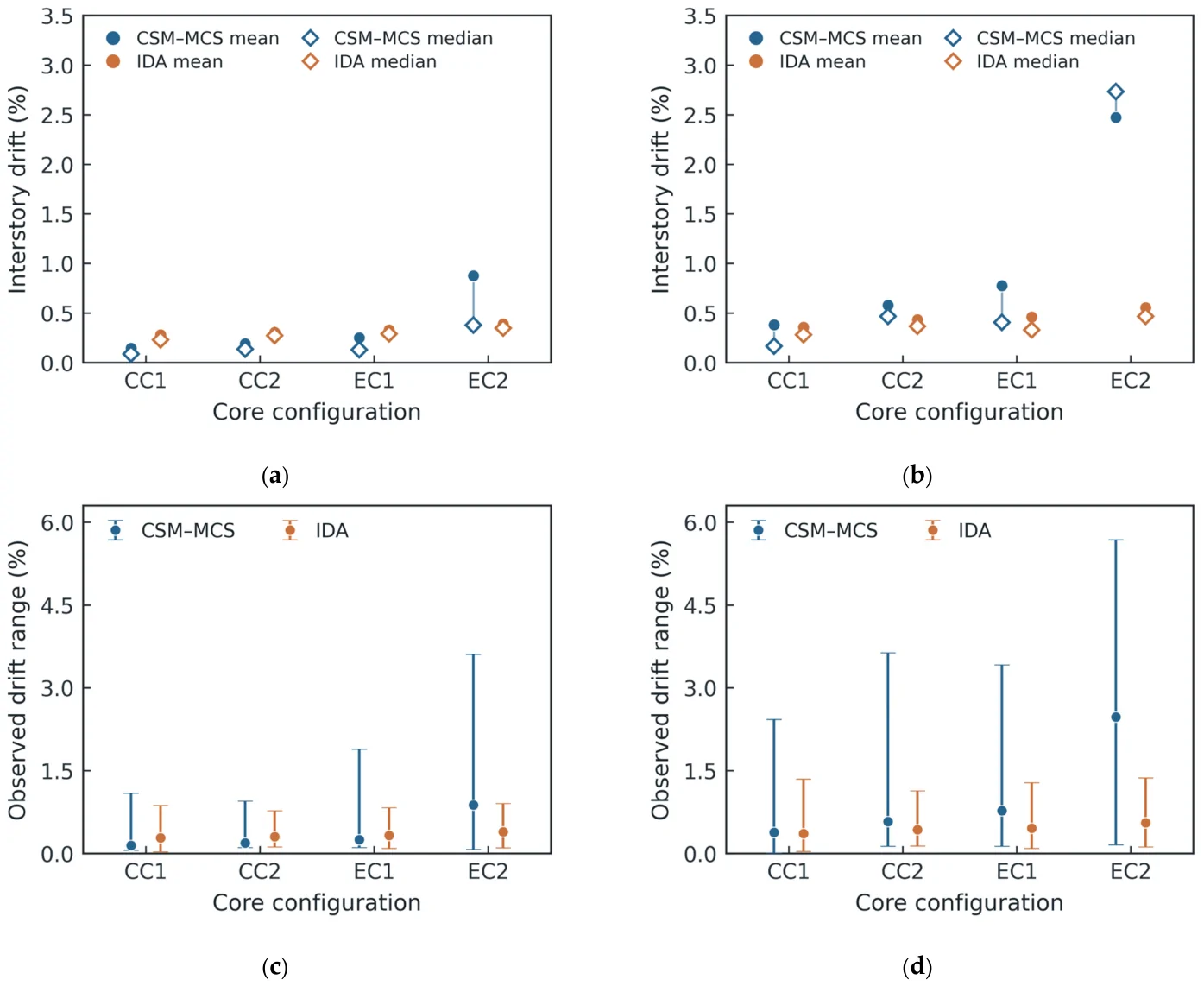
Central drift estimates and response ranges: (**a**) means and medians at 0.598 g; (**b**) means and medians at 0.702 g; (**c**) ranges at 0.598 g; (**d**) ranges at 0.702 g. Filled circles denote means; open diamonds denote medians. Whiskers show observed minimum–maximum ranges.

Mean-based ordering is CC1 < CC2 < EC1 < EC2 in the full case ensembles. Median ordering differs: EC1 is below CC2 in CSM–MCS at both levels and in IDA at the higher level. At 0.702 g, the EC1 and CC2 medians are 0.4065% and 0.4680% for CSM–MCS, and 0.3307% and 0.3664% for IDA. Figure 4 places the central estimates within the observed ranges.

The CSM–MCS EC2 mean/median ratio changes from 2.313 to 0.904. At the higher coordinate, its range is 0.1580–5.6850%, compared with 0.1188–1.3682% for IDA. The contrast between mean, median and extrema motivates the distributional comparison in Section 3.5.

### 3.2. Absolute and Relative Dispersion

**Figure 5:**
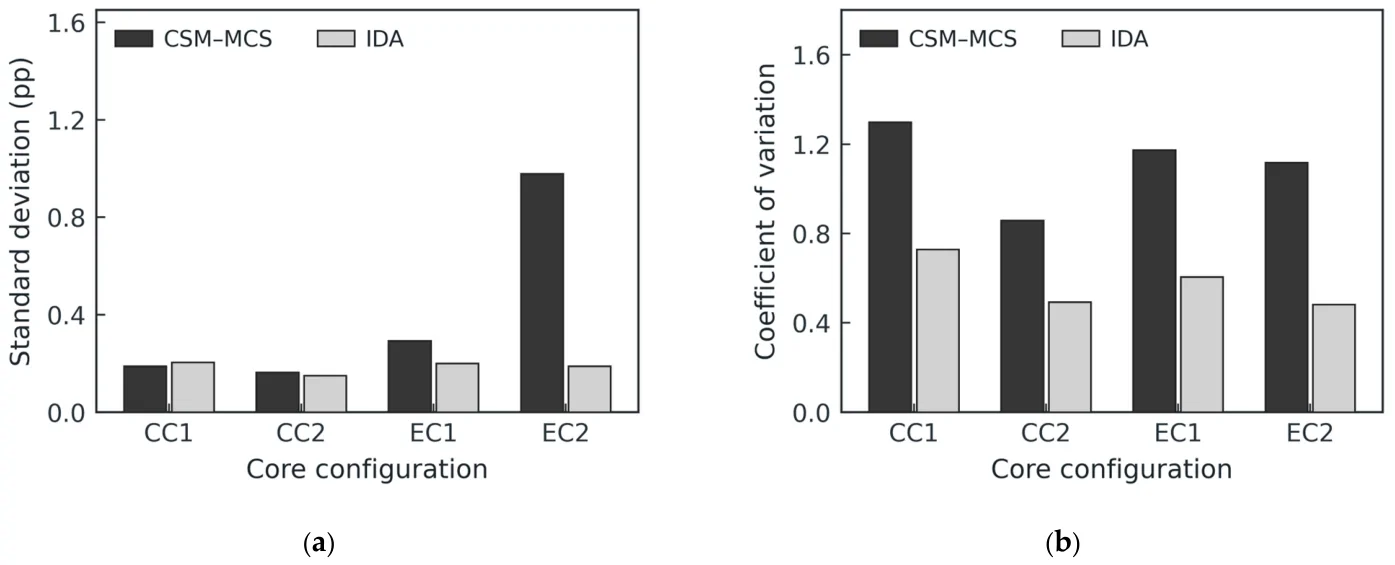

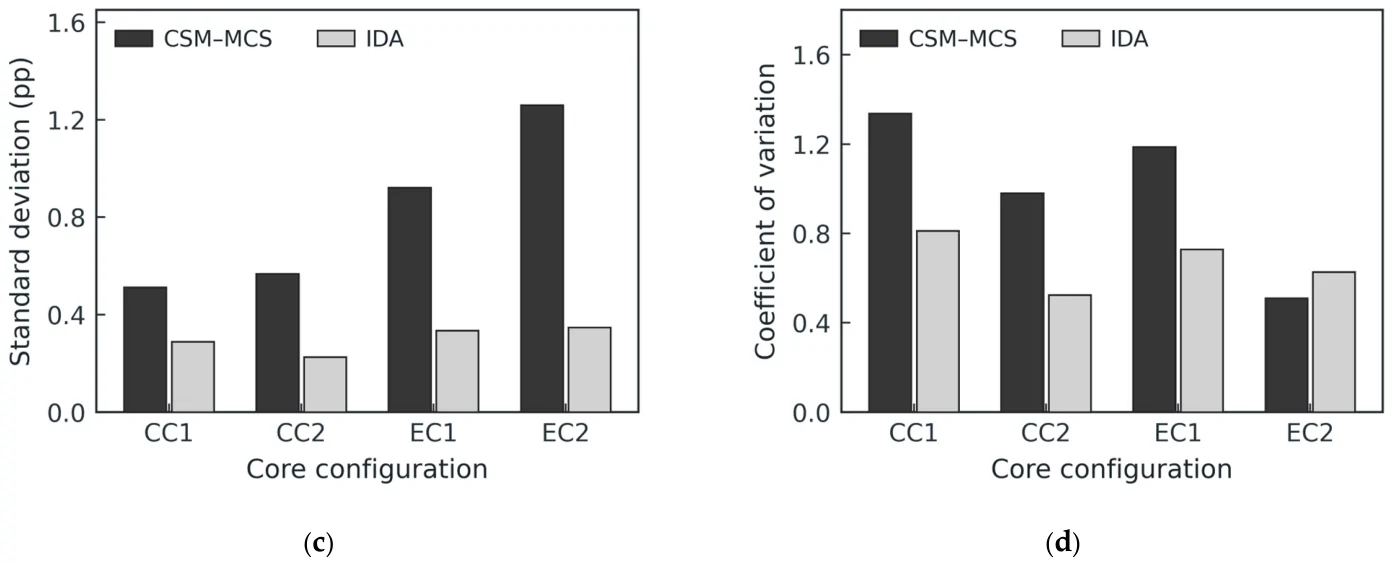
Absolute and relative dispersion: (**a**) standard deviation at 0.598 g; (**b**) COV at 0.598 g; (**c**) standard deviation at 0.702 g; (**d**) COV at 0.702 g. pp denotes percentage points.

CSM–MCS gives EC2 the largest absolute SD at both coordinates. At 0.702 g, its SD is 1.2604 percentage points and its COV is 0.510. CC1 has SD = 0.5124 percentage points and COV = 1.338. Thus, EC2 has about 6.45 times the CC1 mean but only 0.381 times its COV. A small COV can coexist with a large absolute response scale (Figure 5).

This contrast follows from normalization: COV amplification equals SD amplification divided by mean amplification. COV does not provide an independent measure of safety. In IDA, EC2 has a smaller SD than CC1 at the lower coordinate and a larger SD at the higher coordinate, while its COV remains smaller at both. Absolute and relative dispersion therefore retain different information.

### 3.3. Contrasts Between Procedures

**Figure 6:**
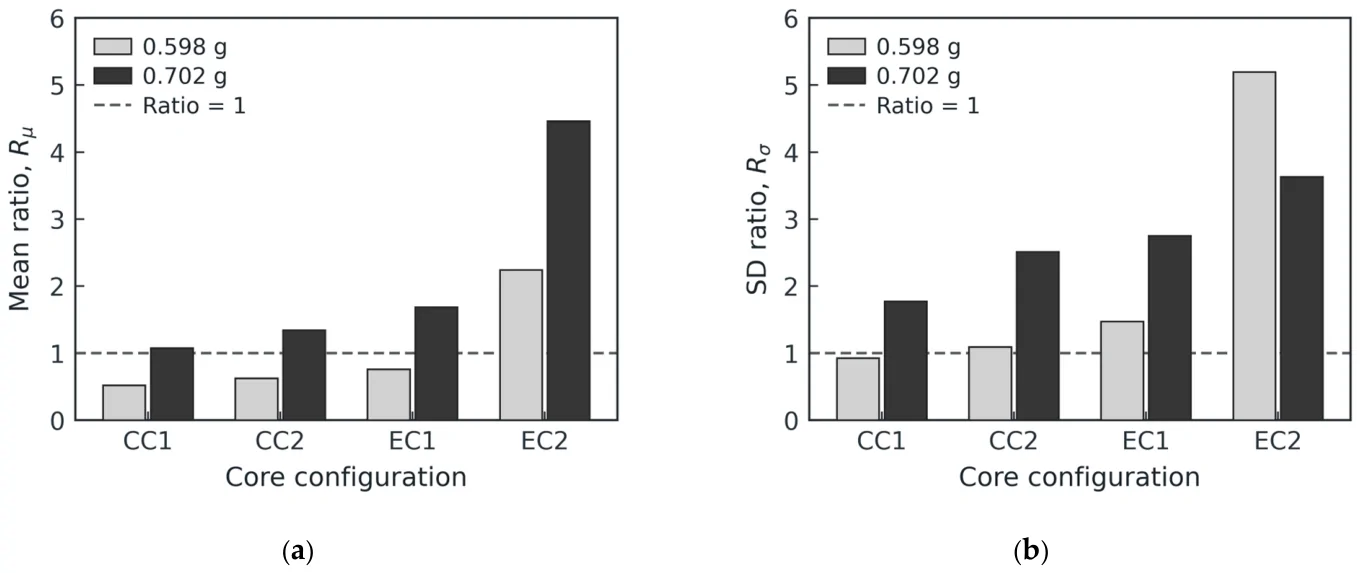
Between-procedure ratios for (**a**) mean drift *R_μ_* and (**b**) standard deviation *R_σ_*. The dashed line denotes unity. Ratios compare the complete conditional implementations.

At 0.598 g, CSM–MCS-to-IDA mean ratios are 0.518, 0.625, 0.759 and 2.242 for CC1, CC2, EC1 and EC2. At 0.702 g, all four exceed unity: 1.074, 1.343, 1.686 and 4.462 (Table 8; Figure 6). Three comparisons change direction across the interval. These contrasts characterize the simultaneous material, model, excitation and damping differences in Table 3.

For EC2, the absolute-SD ratio decreases from 5.195 to 3.628 while the mean ratio increases. Central demand and spread therefore cannot be represented by the same scalar trend. The ratios do not identify the dominant uncertainty source or establish a ranking of method accuracy.

### 3.4. Configuration and Intensity Amplification

Table 9 normalizes mean and SD to CC1. Every non-reference case has a larger mean, while some IDA SD amplifications are below one. CC2, for example, has smaller absolute SD than CC1 at both levels. A consistent mean order does not imply the same order of spread.

**Figure 7:**
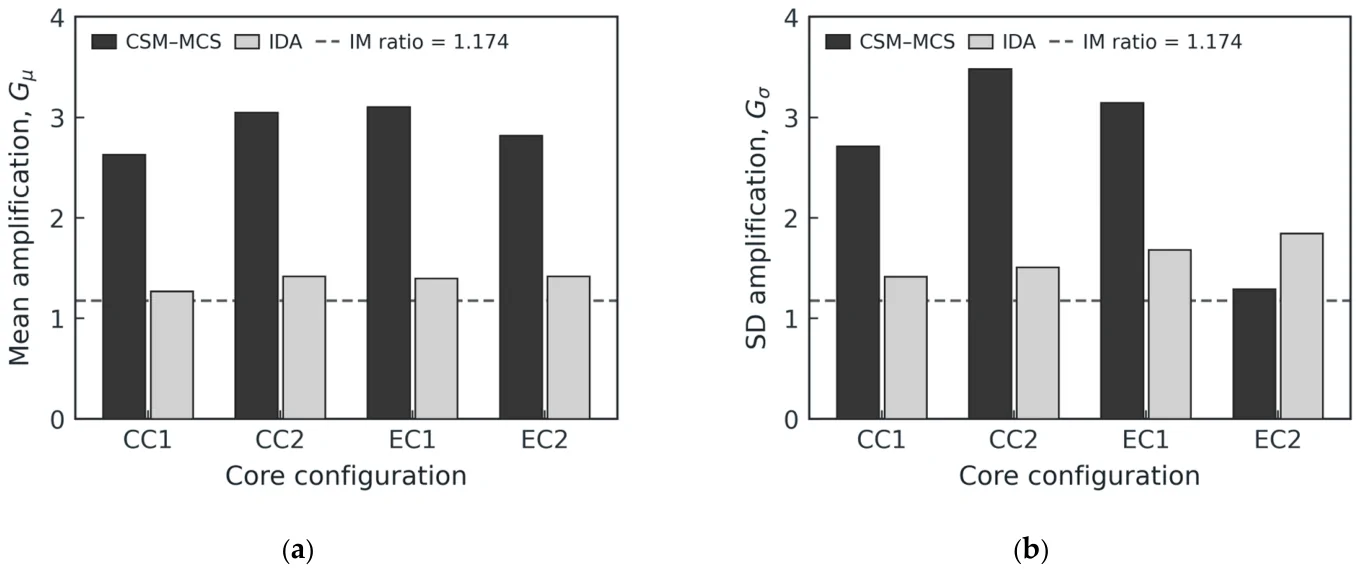
Finite amplification from 0.598 to 0.702 g: (**a**) mean drift *G_μ_*; (**b**) standard deviation *G_σ_*. The dashed line denotes the IM ratio of 1.174, included in the legend. Both vertical axes extend to 4.0.

The IM coordinate increases by 0.702/0.598 = 1.174. Mean drift grows by factors of 2.628–3.103 for CSM–MCS and 1.268–1.418 for IDA (Table 10; Figure 7). CSM–MCS EC2 has SD growth of approximately 1.288 and mean growth of 2.820, giving COV growth near 0.457. IDA EC2 has increasing relative dispersion over the same interval.

**Figure A3:**
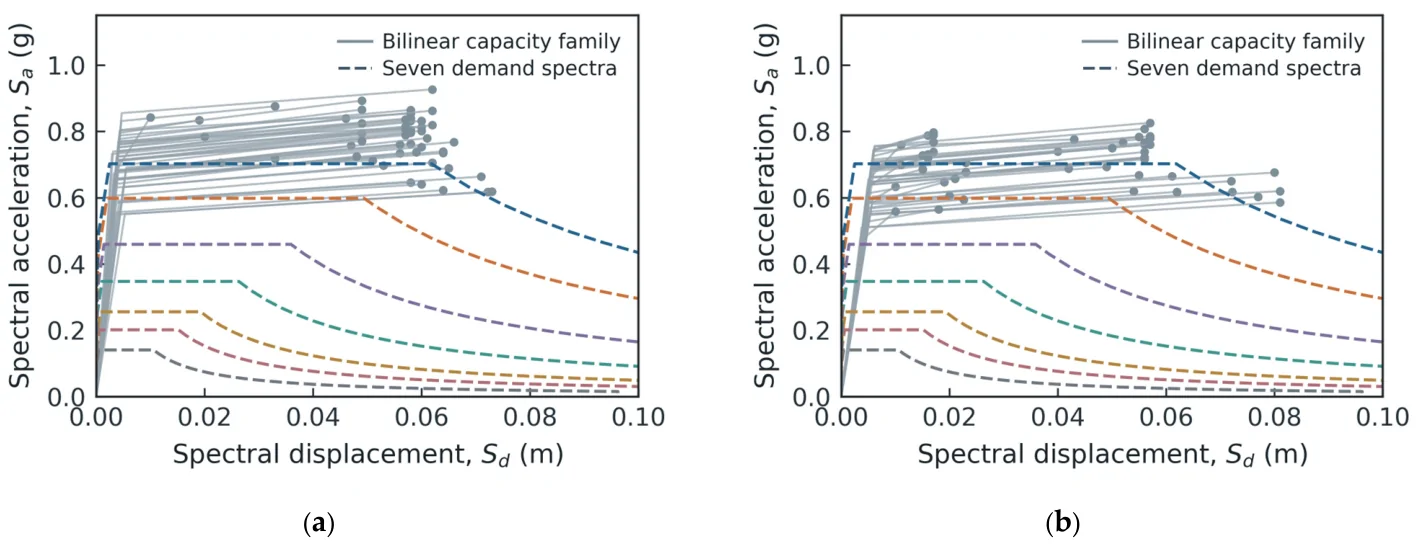

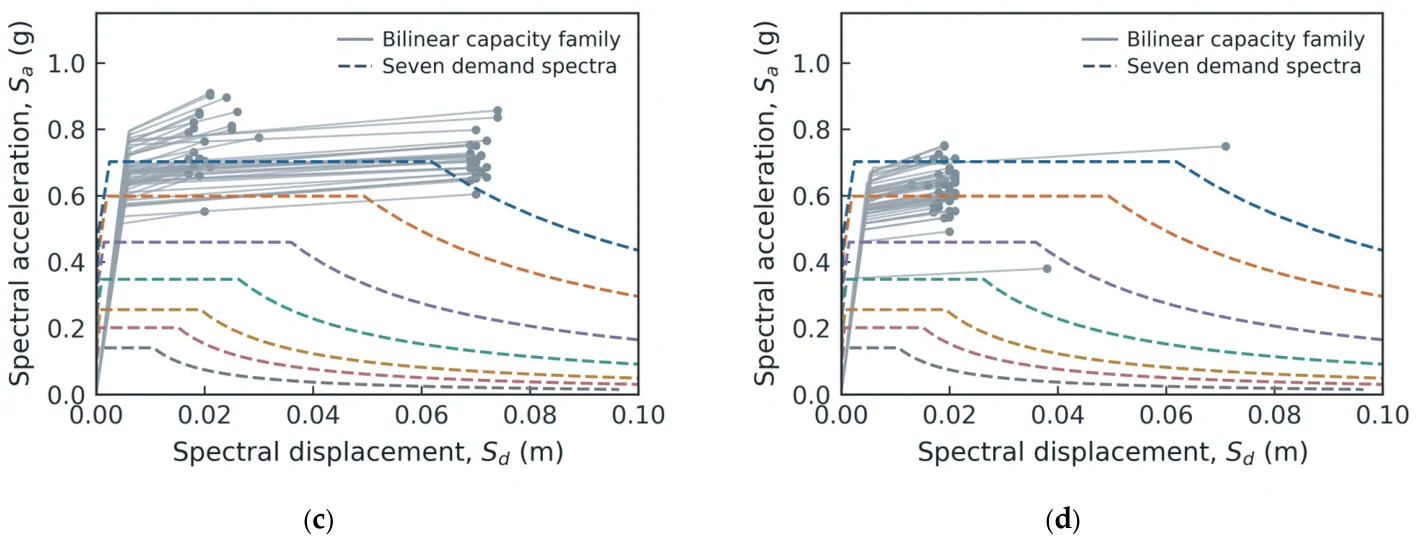
Capacity–demand diagrams for (**a**) CC1, (**b**) CC2, (**c**) EC1 and (**d**) EC2, redrawn from the numerical chart series [16]. Gray solid curves with circular endpoints represent the bilinear capacity family. The dashed demand spectra correspond, from bottom to top, to return-period labels of 50 (gray), 100 (rose), 200 (ochre), 500 (teal), 1000 (purple), 2400 (orange) and 4800 (blue) years. Panel names follow the nominal geometry labels (Appendix C).

**Figure A4:**
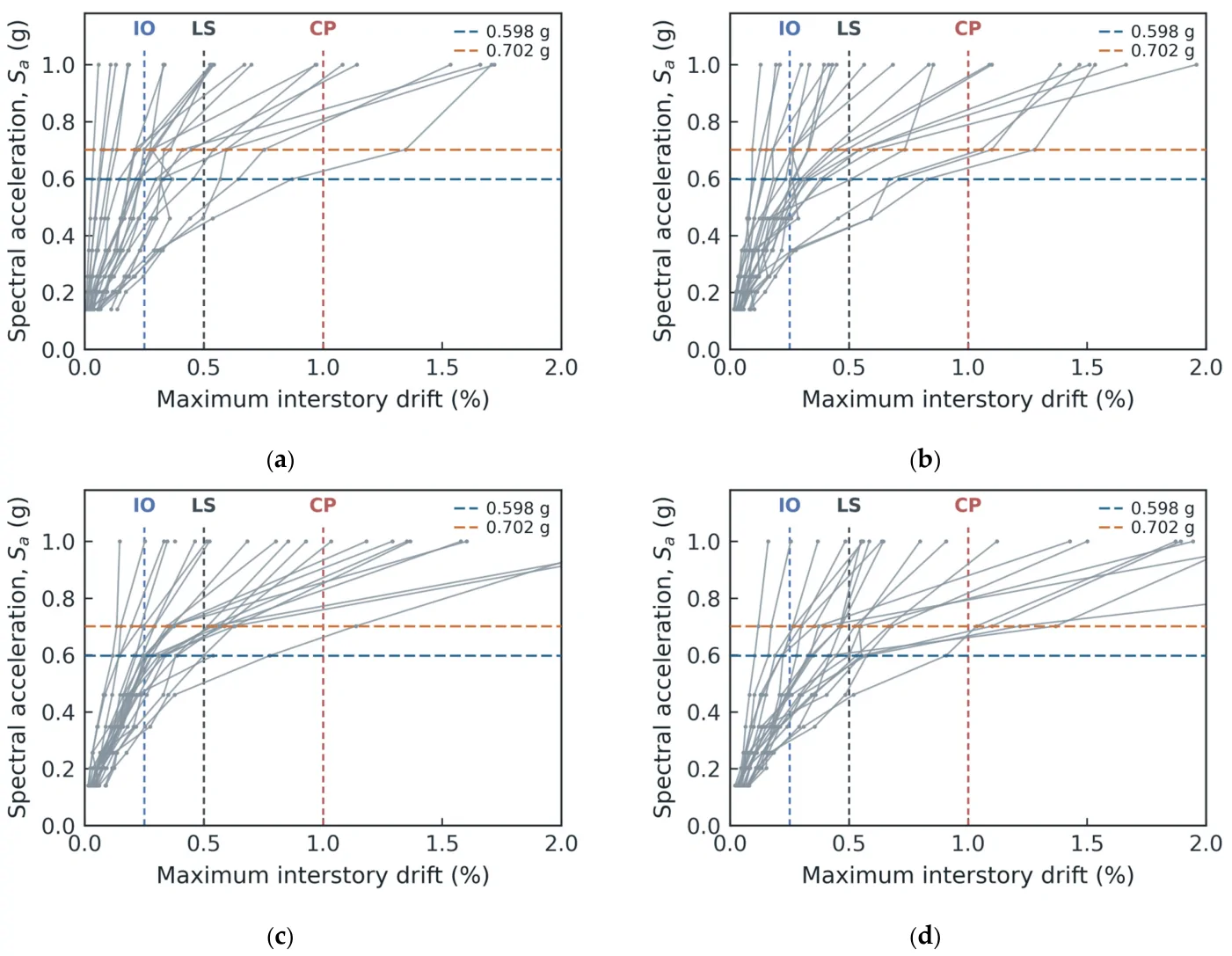
IDA curves for (**a**) CC1, (**b**) CC2, (**c**) EC1 and (**d**) EC2 [16], redrawn from available coordinates using the nominal geometry labels (Appendix C). Gray lines and circular markers represent individual record-case IDA responses. Markers indicate stored coordinates; missing coordinates remain unconnected. IO, LS and CP denote immediate occupancy, life safety and collapse prevention reference levels; they are not acceptance limits adopted by this article.

Supplementary File S1 provides intermediate-coordinate statistics for 28 CSM–MCS and 32 IDA cases, extending the numerical description of growth. Figure A3 and Figure A4 supply the capacity–demand and IDA context. These finite increments quantify response growth; identifying an instability threshold or a damping-induced transition would additionally require realization-specific intersections and state histories.

### 3.5. Empirical Distributions and Upper Percentiles

**Figure 8:**
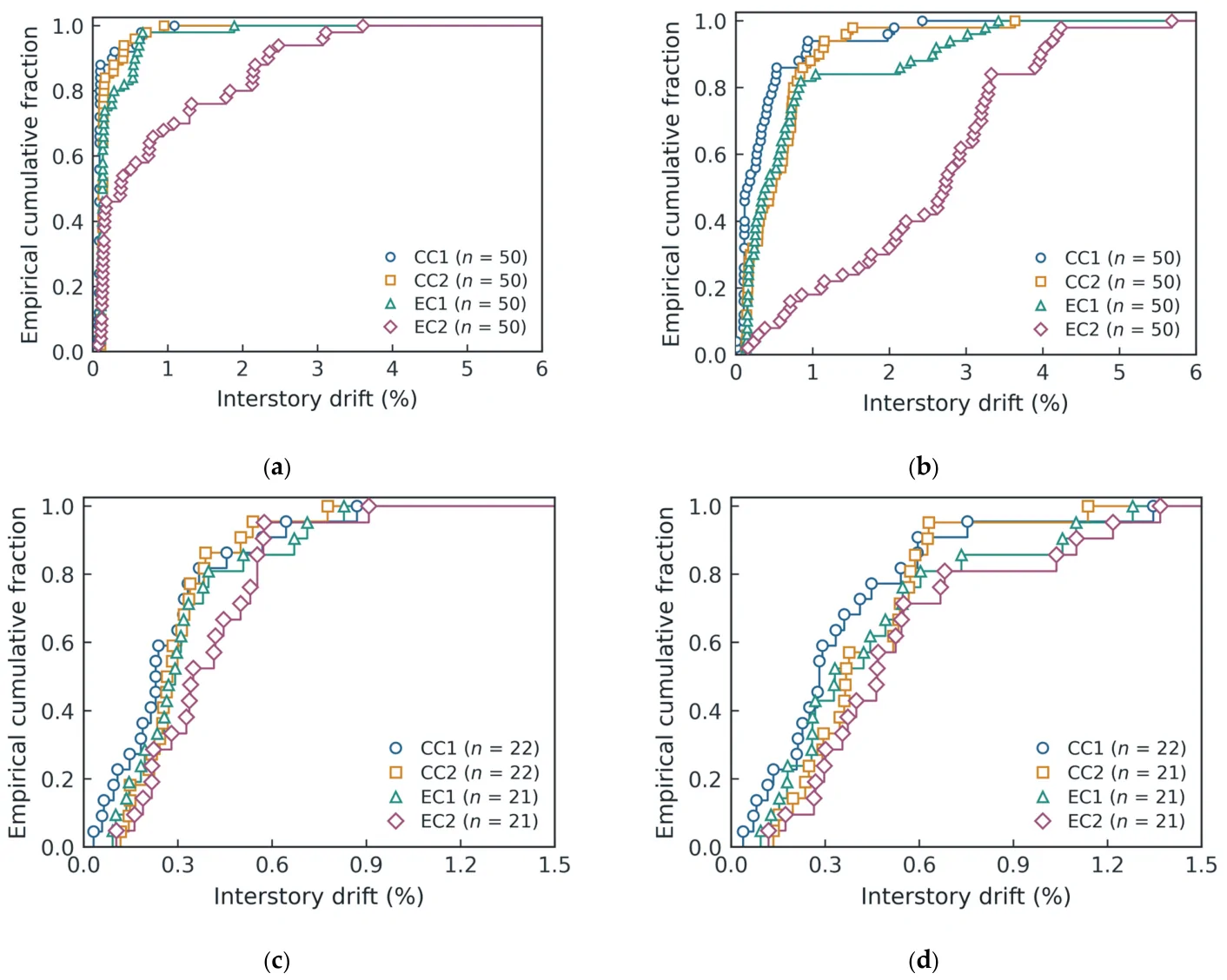
Empirical cumulative response distributions: (**a**) CSM–MCS at 0.598 g; (**b**) CSM–MCS at 0.702 g; (**c**) IDA at 0.598 g; (**d**) IDA at 0.702 g. Legends give the number of entries. Step lines show the empirical distributions; open markers identify actual response coordinates. No smoothing or parametric density fit is used.

Table 11 and Figure 8 extend the comparison to empirical distributions. At 0.702 g, IDA CC1 has a smaller mean than CC2, but *Q*_95_ is 0.7451% for CC1 and 0.6299% for CC2. The mean ordering reverses at this upper sample percentile. EC1 and EC2 have *Q*_95_ values of 1.1000% and 1.2172%. This identifies a distinction absent from a mean-based ordering.

The CSM–MCS EC2 distribution also changes unevenly across percentiles. Its median increases from 0.3790% to 2.7335%, *Q*_84_ from 2.1364% to 3.4214%, and *Q*_95_ from 2.8061% to 4.1609%. Changes in the center and upper tail are not proportional, explaining why mean growth, absolute-SD growth and COV growth convey different distributional information.

### 3.6. Listed Record Groups and Response Influence

Table 12 compares the near and far groups defined by the selection list. At 0.702 g, EC2 has near/far means of 0.6131%/0.4888%, while CC1 has 0.3499%/0.3634%. The far/near mean ratio is approximately 0.797 for EC2 and 1.039 for CC1. The same listed subset does not give the larger mean in every configuration.

The grouping follows the intended list and Section 2.1. Because the records differ in more than distance or pulse classification, this comparison evaluates contributions to the selected suite rather than isolating a near-field or directivity effect.

One-record and one-listed-event omission calculations quantify influence on the mean and COV (Table 13). The largest high-intensity CC1 entry contributes about 17.15% of the response sum. Event omission shifts mean and COV by different amounts, identifying comparisons that depend on influential records. Original entries remain in the baseline ensembles; omissions serve a diagnostic purpose.

### 3.7. Common Records and Configuration Ordering

The intersection of nonmissing IDs contains 20 records at 0.598 g and 19 at 0.702 g. On these common sets, the baseline mean order remains CC1 < CC2 < EC1 < EC2 (Table 14). The baseline ordering is therefore retained when the differing nonmissing record counts are equalized.

Omitting the listed Loma Prieta group gives retained counts of 18 and 17 and the order CC1 < EC1 < CC2 < EC2 at both coordinates. The two intermediate cases exchange order. Equalizing record IDs preserves the baseline ordering, but does not make the intermediate comparison insensitive to event composition.

### 3.8. Limits of a Common Proportional Relation

Equation (10) and Table 15 test whether one scale can represent central estimates from both procedures. Across all eight configuration–intensity comparisons, the minimum worst-case mismatch factor is 2.934 for means and 3.947 for medians. Even a scale minimizing the largest discrepancy leaves substantial case-to-case mismatch.

The bound depends on the intensity set and central statistic. It measures consistency among the numerical ratios rather than prediction error against observations. Together with the distributions and record sensitivities, it identifies information lost when the comparison is reduced to one multiplier.

## 4. Discussion

### 4.1. Choice of Statistic and Stability of Rankings

The combined results show that comparative judgments depend on the response statistic and the included records. A lower COV can accompany larger absolute drift, a lower mean can accompany a larger upper percentile, and a baseline mean ordering can change after a listed event is omitted. These observations concern different assessment questions and should be reported with their numerical populations.

The high-intensity CC1–CC2 *Q*_95_ reversal matters when a comparison is intended to represent large demands rather than average response. Conversely, *Q*_95_ in an ensemble of 21 or 22 entries depends on only a few order statistics. Reporting it with the mean and record influence makes that comparison explicit without turning a sample percentile into a reliability guarantee.

Common-record comparison separates unequal nonmissing counts from record-composition effects. The Loma Prieta omission identifies a specific sensitivity within the common set. Several stations share an earthquake, so listed-event influence differs from one-record influence. This distinction is retained without assuming that the stations constitute independent earthquake observations.

### 4.2. Structural Interpretation and Damping

Core position changes the lever arm between the centers of mass and lateral resistance. Translation can be accompanied by floor rotation, and yielding can redistribute stiffness and local demand [4,5]. The directional descriptors and reported capacity shapes provide context for distinct core arrangements. Quantifying a configuration-specific torsional contribution would additionally require confirmed model-to-response correspondence and directional modal participation, rotation and plan-edge drift histories.

The capacity–demand diagrams show how a prescribed demand increase can move an intersection toward a different part of a nonlinear capacity curve. This provides a plausible explanation for large finite static increments. The intermediate-coordinate statistics add numerical context, while realization-specific branch selection and intersection paths would be required to establish the mechanism of a particular response transition.

The damping defining *S*_a_(*T*_1_, 5%) differs from effective damping in equivalent-linearization displacement estimation [12,13]. The static population here is defined by prescribed 5%-damped demand spectra. Replacing that demand model would define an additional response population and require corresponding performance-point calculations. The present ratios are therefore properties of the documented implementations rather than a general correction for iterative CSM formulations.

### 4.3. Assessment Implications

Extended IDA [10] and FRACAS [15] demonstrate ways of representing additional uncertainty populations. Their formulations also clarify why SDs from differently conditioned ensembles cannot be used to recover separate material and record variance contributions. North American collapse assessment of ductile RC frames [11] addresses a different structural population and performance objective. The present comparisons instead describe how the two specified piloti demand ensembles differ in magnitude, dispersion and proportional relationship.

For assessment comparisons, the findings favor reporting absolute demand together with relative dispersion and upper sample percentiles. Closely spaced means should be accompanied by record-composition sensitivity before they are used to establish a configuration preference. The common-scale mismatch shows why one procedure-to-procedure multiplier would hide configuration and intensity dependence in these ensembles.

A structural performance decision additionally requires defined objectives and appropriate acceptance quantities. The American Society of Civil Engineers/Structural Engineering Institute (ASCE/SEI) standard 41-23 provides an existing-building assessment framework [27], while ASCE/SEI 7-22 and the International Building Code use their own demand definitions and requirements [28,29]. Building-level drift statistics complement, rather than replace, checks of ground-story column axial load, shear and rotation and, where torsion is important, plan-edge deformation. Damage exceedance, code compliance and retrofit decisions require those performance criteria and response quantities.

### 4.4. Applicability and Conditions of Interpretation

The findings concern one archetype, four nominal configuration datasets and two conditional analysis populations. Material treatment, member idealization, excitation and damping change together between procedures. The study therefore addresses differences between complete implementations, rather than decomposing them into independent causal contributions. The listed motions form a mixed benchmark subset rather than a site-specific hazard calibration.

Appendix C records material correspondence conditions: intermediate model-label mapping, summary/export discrepancies, omitted records and an extreme output of unclassified status, IM-folder differences, motion-identity exceptions and three formula-linked CSM entries. These conditions limit physical attribution even though the entry-level arithmetic is reproducible. The results are consequently conditional descriptive comparisons, pending the checks required for verified configuration-specific structural conclusions.

A controlled extension could separate damping, constitutive-model and uncertainty effects using a common model and matched inputs. Verification of the detailed structural and solver settings listed in Appendix C would support that extension. Component-level acceptance quantities and suitable benchmarks would then connect the comparative indicators to a performance or retrofit decision.

For statistical inference, the sampling design, paired components, repeated events and treatment of unsuccessful analyses must be specified together. Here, percentiles describe the empirical ensembles, omissions quantify deterministic influence, and the proportional bound is an arithmetic result. This separation gives each additional comparison a defined meaning without claiming population confidence coverage or universal convergence.

## 5. Conclusions

The study integrates central demand, absolute and relative dispersion, empirical distributions, record-composition sensitivity and proportional consistency. The resulting findings for the two conditional analysis populations are as follows.

1. EC2 has the largest mean in the compared ensembles. Its mean amplification relative to CC1 is 6.01–6.45 for fixed-demand-damping CSM–MCS and 1.39–1.55 for IDA. These contrasts characterize the complete implementations.
2. At 0.702 g, EC2 combines the largest static SD with the smallest COV. A smaller normalized dispersion does not imply smaller absolute demand; mean, SD and COV must be interpreted jointly.
3. Empirical percentiles add information beyond the mean order. At the higher coordinate, IDA CC1 has a smaller mean but a larger *Q*_95_ than CC2. Static EC2 has different growth at the median, *Q*_84_ and *Q*_95_, showing unequal changes across its distribution.
4. Common-record sets preserve the baseline IDA mean order, while omission of the listed Loma Prieta group reverses CC2 and EC1 at both coordinates. The comparison therefore depends on the response statistic and record composition. This is a deterministic influence result.
5. Across the eight configuration–intensity comparisons, any common proportional scale leaves minimum worst-case mismatch factors of approximately 2.93 for means and 3.95 for medians. A single multiplier cannot closely represent the central estimates across the compared cases.

The combined results support reporting response scale, upper sample percentiles and record composition alongside normalized dispersion. They provide a more informative comparison of the documented implementations than a mean ranking or one multiplier alone. Application to verified physical configurations and structural performance decisions remains subject to Appendix C and appropriate component acceptance quantities.

## Figures and Tables

**Table 1:** Adopted analysis basis and additional comparisons in this article.

Assessment Aspect	Adopted Analysis Basis [16]	Additional Comparison in This Article
Central demand and spread	Four configurations; CSM–MCS and IDA response entries	Recalculate means, medians, sample SD, COV and ranges with explicit counts
Distribution and intensity	Individual material/record responses across IM coordinates	Compare empirical distributions, percentiles and unequal center/tail growth
Record composition	Numbered cases and the intended event–station list	Compare near/far groups, common IDs and record/event influence
Proportionality	Different material, excitation and model conditions	Derive a common-scale mismatch bound and define its interpretation

**Table 2:** Core-layout descriptors.

Layout	*R*_eX_	*R*_eY_	Configuration
CC1	0.04	0.00	Nearly centered
CC2	0.48	0.01	Predominantly one-direction eccentricity
EC1	0.41	0.65	Eccentric in both normalized directions
EC2	0.38	0.87	Largest *R*_eY_ of the four layouts

**Table 3:** Analysis conditions for the two implementations.

Feature	CSM–MCS	IDA
Nonlinear idealization	Beam, column and wall concentrated hinges	Beam hinges; column and wall fibers
Strength inputs	Sampled *f*_c_ and *f*_y_	Nominal *f*_c_ = 21 MPa; *f*_y_ = 400 MPa
Population	50 indexed material entries per case	22 listed pairs; 21–22 nonmissing entries per principal case
Demand	Prescribed ADRS spectra	Amplitude-scaled acceleration histories
Damping	Fixed 5% demand damping	Reported 5% Rayleigh damping
Solution	Bilinear capacity–demand intersections	Newmark integration and modified Newton iteration
Scope	Four-story reported archetype; Section 2.1 and Appendix C define correspondence	Same nominal archetype; Section 2.1 and Appendix C define correspondence

**Table 4:** Static intensity coordinates and return-period labels.

Return-Period Label (Years)	50	100	200	500	1000	2400	4800
*S*_a_(*T*_1_, 5%) (g)	0.141	0.202	0.256	0.348	0.460	0.598	0.702

**Table 5:** Material-strength assumptions in the static probabilistic branch.

Strength Variable	Distribution	Arithmetic Mean (MPa)	SD (MPa)	COV
*f*_c_	Normal	21	3.78	0.180
*f*_y_	Lognormal	400	39.2	0.098

**Table 6:** CSM–MCS drift statistics for the 50 indexed entries.

*S*_a_ (g)	Layout	*n*	Mean (%)	Median (%)	SD (pp)	COV	Min (%)	Max (%)
0.598	CC1	50	0.1458	0.0865	0.1891	1.297	0.0570	1.0890
0.598	CC2	50	0.1903	0.1355	0.1633	0.858	0.1080	0.9510
0.598	EC1	50	0.2498	0.1305	0.2929	1.172	0.1060	1.8900
0.598	EC2	50	0.8765	0.3790	0.9784	1.116	0.0768	3.6070
0.702	CC1	50	0.3831	0.1660	0.5124	1.338	0.0110	2.4300
0.702	CC2	50	0.5797	0.4680	0.5680	0.980	0.1280	3.6380
0.702	EC1	50	0.7752	0.4065	0.9208	1.188	0.1320	3.4200
0.702	EC2	50	2.4716	2.7335	1.2604	0.510	0.1580	5.6850

*n* counts indexed entries; the separate nominal reference is excluded. SD is in percentage points (pp). Section 2.1 and Appendix C define the numerical basis.

**Table 7:** IDA drift statistics and nonmissing response counts.

*S*_a_ (g)	Layout	*n*	Mean (%)	Median (%)	SD (pp)	COV	Min (%)	Max (%)
0.598	CC1	22	0.2812	0.2294	0.2046	0.728	0.0314	0.8703
0.598	CC2	22	0.3045	0.2730	0.1499	0.492	0.1174	0.7762
0.598	EC1	21	0.3293	0.2909	0.1993	0.605	0.0923	0.8280
0.598	EC2	21	0.3909	0.3489	0.1884	0.482	0.1040	0.9073
0.702	CC1	22	0.3567	0.2808	0.2891	0.810	0.0379	1.3460
0.702	CC2	21	0.4318	0.3664	0.2262	0.524	0.1349	1.1375
0.702	EC1	21	0.4597	0.3307	0.3347	0.728	0.0938	1.2799
0.702	EC2	21	0.5539	0.4654	0.3474	0.627	0.1188	1.3682

*n* counts nonmissing record cases. Missing and omitted values are not assigned zero. Section 2.1 and Appendix C define the numerical basis.

**Table 8:** Between-procedure ratios of central demand and dispersion.

*S*_a_ (g)	Layout	*R_μ_*	*R_σ_*	*R*_COV_
0.598	CC1	0.518	0.924	1.783
0.598	CC2	0.625	1.089	1.742
0.598	EC1	0.759	1.469	1.937
0.598	EC2	2.242	5.195	2.317
0.702	CC1	1.074	1.773	1.650
0.702	CC2	1.343	2.512	1.871
0.702	EC1	1.686	2.751	1.631
0.702	EC2	4.462	3.628	0.813

*R_μ_*, *R_σ_* and *R*_COV_ compare the conditional implementations. Ratios are calculated before rounding.

**Table 9:** Configuration amplification relative to CC1.

*S*_a_ (g)	Layout	*A_μ_* CSM–MCS	*A_σ_* CSM–MCS	*A_μ_* IDA	*A_σ_* IDA
0.598	CC2	1.306	0.863	1.083	0.733
0.598	EC1	1.714	1.549	1.171	0.974
0.598	EC2	6.013	5.174	1.390	0.920
0.702	CC2	1.513	1.109	1.211	0.782
0.702	EC1	2.024	1.797	1.289	1.158
0.702	EC2	6.452	2.460	1.553	1.202

CC1 equals unity by definition and is omitted. Configuration labels follow Section 2.1.

**Table 10:** Finite amplification between the two nominal coordinates.

Layout	*G_μ_* CSM–MCS	*G_σ_* CSM–MCS	*G*_COV_ CSM–MCS	*G_μ_* IDA	*G_σ_* IDA	*G*_COV_ IDA
CC1	2.628	2.710	1.031	1.268	1.412	1.114
CC2	3.046	3.479	1.142	1.418	1.508	1.064
EC1	3.103	3.144	1.013	1.396	1.679	1.203
EC2	2.820	1.288	0.457	1.417	1.845	1.302

*G*_COV_ = *G_σ_*/*G_μ_*. The nominal IM ratio is 0.702/0.598 = 1.174.

**Table 11:** Empirical response percentiles at the two comparison coordinates.

Procedure	*S*_a_ (g)	Layout	*n*	*Q*_16_ (%)	*Q*_50_ (%)	*Q*_84_ (%)	*Q*_95_ (%)
CSM–MCS	0.598	CC1	50	0.0820	0.0865	0.0982	0.6068
CSM–MCS	0.598	CC2	50	0.1208	0.1355	0.1819	0.4947
CSM–MCS	0.598	EC1	50	0.1200	0.1305	0.5366	0.6240
CSM–MCS	0.598	EC2	50	0.1237	0.3790	2.1364	2.8061
CSM–MCS	0.702	CC1	50	0.1028	0.1660	0.5282	1.5120
CSM–MCS	0.702	CC2	50	0.1514	0.4680	0.8416	1.3060
CSM–MCS	0.702	EC1	50	0.1607	0.4065	1.2160	2.9183
CSM–MCS	0.702	EC2	50	0.8358	2.7335	3.4214	4.1609
IDA	0.598	CC1	22	0.0999	0.2294	0.4232	0.6406
IDA	0.598	CC2	22	0.1687	0.2730	0.3857	0.5363
IDA	0.598	EC1	21	0.1519	0.2909	0.4860	0.7112
IDA	0.598	EC2	21	0.2167	0.3489	0.5527	0.5746
IDA	0.702	CC1	22	0.1231	0.2808	0.5747	0.7451
IDA	0.702	CC2	21	0.2379	0.3664	0.5829	0.6299
IDA	0.702	EC1	21	0.1788	0.3307	0.7074	1.1000
IDA	0.702	EC2	21	0.2745	0.4654	0.9653	1.2172

*Q_p_* denotes a sample percentile obtained by linear interpolation.

**Table 12:** IDA comparison by listed near and far groups.

*S*_a_ (g)	Layout	*n*_near_/*n*_far_	*μ*_near_ (%)	*μ*_far_ (%)	COV_near_	COV_far_	*μ*_far_/*μ*_near_
0.598	CC1	11/11	0.2394	0.3229	0.989	0.518	1.349
0.598	CC2	11/11	0.2953	0.3137	0.649	0.323	1.062
0.598	EC1	11/10	0.2600	0.4056	0.640	0.524	1.560
0.598	EC2	11/10	0.4092	0.3708	0.583	0.327	0.906
0.702	CC1	11/11	0.3499	0.3634	1.079	0.499	1.039
0.702	CC2	11/10	0.4036	0.4628	0.706	0.316	1.147
0.702	EC1	11/10	0.3627	0.5664	0.771	0.656	1.561
0.702	EC2	11/10	0.6131	0.4888	0.702	0.474	0.797

*n*_near_/*n*_far_ gives actual nonmissing counts in the intended near/far groups. Appendix C qualifies applied-input identity.

**Table 13:** IDA record and listed-event omission sensitivities.

*S*_a_ (g)	Layout	Largest Record Share (%)	Δ*μ* One Record (%)	Δ*μ* One Listed Event (%)	COV After Event Omission
0.598	CC1	14.07	−9.98 to 4.23	−13.56 to 4.30	0.649 to 0.763
0.598	CC2	11.59	−7.38 to 2.93	−6.85 to 4.10	0.394 to 0.535
0.598	EC1	11.97	−7.57 to 3.60	−6.45 to 3.35	0.537 to 0.668
0.598	EC2	11.05	−6.61 to 3.67	−6.21 to 4.85	0.421 to 0.524
0.702	CC1	17.15	−13.21 to 4.26	−16.45 to 7.38	0.632 to 0.855
0.702	CC2	12.55	−8.17 to 3.44	−7.81 to 4.47	0.418 to 0.562
0.702	EC1	13.26	−8.92 to 3.98	−7.09 to 3.67	0.650 to 0.795
0.702	EC2	11.76	−7.35 to 3.93	−7.35 to 6.35	0.586 to 0.679

Δ*μ* follows Equation (9), applied to one record or one listed event. Ranges describe deterministic influence; baseline values are retained.

**Table 14:** IDA means on common record IDs and after listed Loma Prieta omission.

*S*_a_ (g)	Record Set	*n*	*μ*_CC1_ (%)	*μ*_CC2_ (%)	*μ*_EC1_ (%)	*μ*_EC2_ (%)	Mean Ordering
0.598	Common IDs	20	0.2859	0.3155	0.3312	0.3996	CC1 < CC2 < EC1 < EC2
0.598	Loma Prieta omitted	18	0.2844	0.3212	0.3091	0.4083	CC1 < EC1 < CC2 < EC2
0.702	Common IDs	19	0.3684	0.4514	0.4805	0.5770	CC1 < CC2 < EC1 < EC2
0.702	Loma Prieta omitted	17	0.3746	0.4603	0.4465	0.6018	CC1 < EC1 < CC2 < EC2

The common sets exclude IDs missing in any configuration. Event grouping follows the intended list.

**Table 15:** Minimum multiplicative mismatch under a common proportional scale.

*S*_a_ (g)	Statistic	*r*_min_	*r*_max_	*c* ^*^	*D*_min_
0.598	Mean	0.518	2.242	1.078	2.080
0.598	Median	0.377	1.086	0.640	1.697
0.702	Mean	1.074	4.462	2.189	2.038
0.702	Median	0.591	5.873	1.863	3.152
Both	Mean	0.518	4.462	1.521	2.934
Both	Median	0.377	5.873	1.488	3.947

Both includes the two coordinates. *r_j_* is the CSM–MCS/IDA central-estimate ratio; c^*^ and *D*_min_ follow Equation (10).

**Figure A2:**
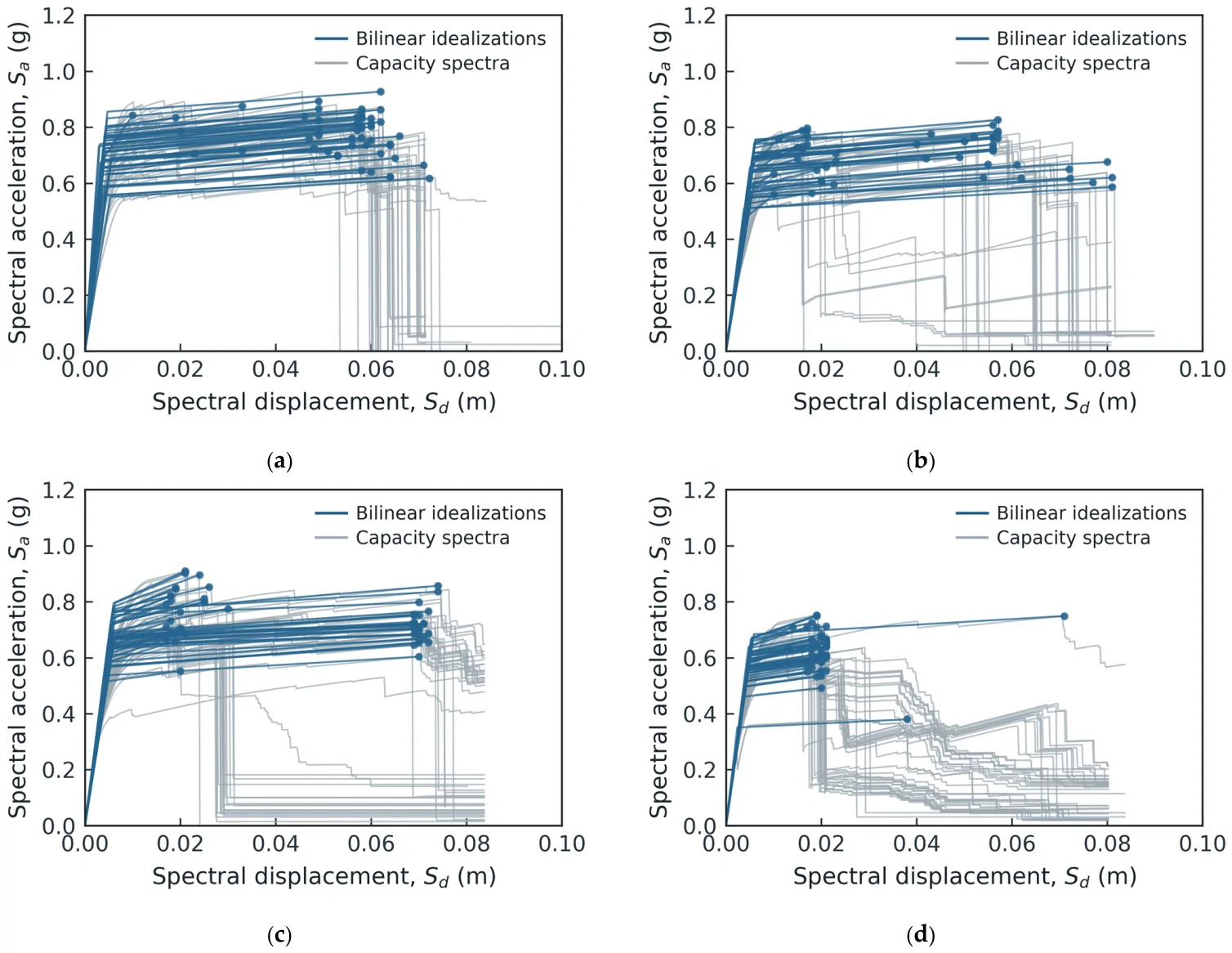
Capacity spectra and bilinear idealizations for (**a**) CC1, (**b**) CC2, (**c**) EC1 and (**d**) EC2, redrawn from the numerical chart series corresponding to [16], Figures 4.23–4.26. Panel names follow the nominal geometry labels; Appendix C specifies their correspondence to the statistical response labels.

## Data Availability

The original contributions presented in this study are included in the article/Supplementary Material. Further inquiries can be directed to the corresponding author.

## Supplementary Materials

The following supporting information can be downloaded at: https://www.mdpi.com/article/10.3390/ma19214723/s1, Table S1: Distribution Statistics; Table S2: Listed Near Far Summaries; Table S3: Influence Ranges; Table S4: Common Record Statistics; Table S5: Common Record Event Sensitivity; Table S6: Proportional Comparison.

## Appendix A. Listed Ground-Motion Suite

Table A1 and Table A2 identify the intended selection list [16,26]. RSN denotes the PEER-NGA record sequence number; P695 ID is the number within the corresponding FEMA set rather than the pooled analysis index. The Cape Mendocino–Rio Dell year follows the metadata (1992). These tables identify listed motions; input-sheet identity conditions are specified in Appendix C.

**Table A1:** Listed near-field pulse-subset records.

P695 ID	Event and Year	Station	*M*	RSN	*V*_s30_ (m/s)	*R*_rup_ (km)
1	Imperial Valley, 1979	El Centro Array #6	6.5	181	203	1.4
2	Imperial Valley, 1979	El Centro Array #7	6.5	182	211	0.6
4	Superstition Hills, 1987	Parachute Test Site	6.5	723	349	1.0
5	Loma Prieta, 1989	Saratoga–Aloha	6.9	802	371	8.5
6	Erzincan, Turkey, 1992	Erzincan	6.7	821	275	4.4
7	Cape Mendocino, 1992	Petrolia	7.0	828	713	8.2
8	Landers, 1992	Lucerne	7.3	879	685	2.2
9	Northridge, 1994	Rinaldi Receiving Station	6.7	1063	282	6.5
10	Northridge, 1994	Sylmar–Olive View	6.7	1086	441	5.3
12	Chi-Chi, Taiwan, 1999	TCU065	7.6	1503	306	0.6
14	Düzce, Turkey, 1999	Düzce	7.1	1605	276	6.6

**Table A2:** Listed far-field records.

P695 ID	Event and Year	Station	*M*	RSN	*V*_s30_ (m/s)	*R*_rup_ (km)
1	Northridge, 1994	Beverly Hills–Mulholland	6.7	953	356	17.2
2	Northridge, 1994	Canyon Country–WLC	6.7	960	309	12.4
3	Düzce, Turkey, 1999	Bolu	7.1	1602	326	12.0
5	Imperial Valley, 1979	Delta	6.5	169	275	22.0
6	Imperial Valley, 1979	El Centro Array #11	6.5	174	196	12.5
8	Kobe, Japan, 1995	Shin-Osaka	6.9	1116	256	19.2
13	Loma Prieta, 1989	Capitola	6.9	752	289	15.2
14	Loma Prieta, 1989	Gilroy Array #3	6.9	767	350	12.8
16	Superstition Hills, 1987	El Centro Imperial County	6.5	721	192	18.2
17	Superstition Hills, 1987	Poe Road temporary	6.5	725	208	11.2
18	Cape Mendocino, 1992	Rio Dell Overpass	7.0	829	312	14.3

## Appendix B. Reported Spectral and Nonlinear Response Diagrams

These diagrams reproduce the spectral, capacity and IDA context in [16]. They complement the numerical comparisons; response entries used in the new calculations are taken from numerical cells rather than reconstructed from plotted pixels.

## Appendix C. Numerical Correspondence and Analysis Eligibility

Table A3 specifies the correspondence and eligibility conditions for the numerical comparisons. Supplementary File S1 retains entries and formula flags. Agreement between a summary cell and a directional maximum establishes numerical compatibility at that cell; structural identity, monitoring location and applied input require their own correspondence checks.

**Table A3:** Numerical correspondence and analysis eligibility conditions.

Condition	Evidence and Numerical Treatment
Configuration correspondence	Center-2/Cp response statistics match EC1 and eccentric-1/E1 statistics match CC2. Geometry descriptors retain their original names. Physical geometry-to-response correspondence for these intermediate cases remains unverified.
Eligibility and counts	CSM uses 50 indexed columns, excluding the nominal reference formerly included in low-intensity CC2. IDA uses 21 or 22 nonmissing entries. Missing and omitted values are not zeros; no values are adjusted to impose monotonicity or an ordering.
Direct-output compatibility	Among 171 principal-coordinate IDA entries, 106 agree with an available directional story maximum (including matching backups), 45 lack a supplied corresponding export, and 20 do not match a supplied directional maximum. Compatibility does not establish final model/input identity.
Omitted and extreme cases	EC2 record 15 has direct exports of approximately 10^97^–10^98^ and is absent from its summary; the cause is unclassified. Center-2 record 19 is omitted despite small finite exports. Eccentric-1 record 22 is missing at the higher coordinate. The recorded eligibility pattern is retained without assigning a collapse/failure cause.
Intensity correspondence	E1 export folders use 0.586/0.708 g labels while worksheets use 0.598/0.702 g. This is a candidate matching rule rather than verified applied intensity. CC1 direct exports at the principal coordinates are absent.
Formula-linked entries	The EC1 sheet contains neighboring-value +0.03 formulas at X31–X33, including shared-formula propagation. Values and flags are retained; they are not certified as direct solver outputs. Smaller-*n* generation/plotting files are excluded from convergence evidence.
Input-function identity	Preparation-sheet 8-1/8-2 Northridge sequences are identical and both match source 8210631. Its 9-1/9-2 Sylmar labels match 8211651/8211652 (Kocaeli–Izmit). Actual solver use of this preparation version is unverified; listed-group/event calculations retain the intended map.
Structural reconstruction	Final reinforcement/confinement, stiffness modifiers, support/diaphragm and P–Δ settings, detailed hinge/fiber laws, damping calibration, software build, tolerances, control-point mappings, failure and intersection-selection rules are not all independently verified. Postprocessing reproducibility is distinguished from a complete structural rerun.
